# Targeting macrophage autophagy for inflammation resolution and tissue repair in inflammatory bowel disease

**DOI:** 10.1093/burnst/tkad004

**Published:** 2023-05-04

**Authors:** Er-jin Wang, Ming-Yue Wu, Zheng-yu Ren, Ying Zheng, Richard D Ye, Chris Soon Heng TAN, Yitao Wang, Jia-Hong Lu

**Affiliations:** State Key Laboratory of Quality Research in Chinese Medicine, Institute of Chinese Medical Sciences, University of Macau, Macao SAR, 999078, China; Center for Metabolic Liver Diseases and Center for Cholestatic Liver Diseases, Department of Gastroenterology, The First Affiliated Hospital (Southwest Hospital), Third Military Medical University (Army Medical University), Chongqing, 400038, China; State Key Laboratory of Quality Research in Chinese Medicine, Institute of Chinese Medical Sciences, University of Macau, Macao SAR, 999078, China; State Key Laboratory of Quality Research in Chinese Medicine, Institute of Chinese Medical Sciences, University of Macau, Macao SAR, 999078, China; Kobilka Institute of Innovative Drug Discovery, School of Life and Health Sciences, The Chinese University of Hong Kong, Shenzhen, 518172, China; Department of Chemistry, College of Science, Southern University of Science and Technology, Shenzhen, 518055, China; State Key Laboratory of Quality Research in Chinese Medicine, Institute of Chinese Medical Sciences, University of Macau, Macao SAR, 999078, China; State Key Laboratory of Quality Research in Chinese Medicine, Institute of Chinese Medical Sciences, University of Macau, Macao SAR, 999078, China; Guangdong-Hong Kong-Macau Joint Lab on Chinese Medicine and Immune Disease Research, University of Macau, China

**Keywords:** Autophagy, Inflammatory bowel diseases, Inflammation, Tissue repair, Efferocytosis, Macrophage

## Abstract

Inflammatory bowel disease (IBD) is a chronic, non-specific, recurrent inflammatory disease, majorly affecting the gastrointestinal tract. Due to its unclear pathogenesis, the current therapeutic strategy for IBD is focused on symptoms alleviation. Autophagy is a lysosome-mediated catabolic process for maintaining cellular homeostasis. Genome-wide association studies and subsequent functional studies have highlighted the critical role of autophagy in IBD via a number of mechanisms, including modulating macrophage function. Macrophages are the gatekeepers of intestinal immune homeostasis, especially involved in regulating inflammation remission and tissue repair. Interestingly, many autophagic proteins and IBD-related genes have been revealed to regulate macrophage function, suggesting that macrophage autophagy is a potentially important process implicated in IBD regulation. Here, we have summarized current understanding of macrophage autophagy function in pathogen and apoptotic cell clearance, inflammation remission and tissue repair regulation in IBD, and discuss how this knowledge can be used as a strategy for IBD treatment.

Highlights

- Mutations in autophagy-related genes are associated with increased risk of IBD.
- Induced autophagy of intestinal macrophages is of great significance in enhancing the clearance of apoptotic cells, promoting inflammation resolution and tissue repair.
- Targeted and controllable regulation of macrophage autophagy at the site of intestinal inflammation is a potential strategy for the treatment of IBD in the future.

## Background

Inflammatory bowel disease (IBD) is a chronic inflammatory disorder of the gastrointestinal tract with unknown etiology that has been divided into Crohn’s disease (CD) and ulcerative colitis (UC) [1]. Clinically, IBD patients usually have symptoms of diarrhea, abdominal pain, blood in the stool and weight loss. In the past decade, the incidence of IBD has shown a rapid upward trend, especially in developed countries [2,3]. To date, the pathogenesis of IBD has been associated with different factors, such as genetic susceptibility, environmental stimulation, intestinal flora triggering and immune dysfunction. It is generally believed that an abnormal immune system and an inflammatory response in the intestinal system play important roles in the occurrence and development of IBD [4,5]. In addition, some environmental, genetic and infectious factors, as well as disturbances of the gut microbiota homeostasis, may be factors that contribute to the persistence of the pathological state [6–11].

Autophagy, a conserved process in eukaryotic evolution, is an important pathway for cells to degrade damaged organelles, folded proteins and other substances through lysosomes. It is divided into microautophagy, macroautophagy and chaperone-mediated autophagy. Macroautophagy is the most widely studied type of autophagy. Unless otherwise mentioned, all autophagy described in this review is macroautophagy. Autophagy occurs throughout the life of cells and can be further induced by conditions such as starvation and inflammation. The process of autophagy generally includes initiation (the formation of double-membrane autophagosomes), elongation, maturation (the binding of autophagosomes and lysosomes) and cargo degradation. Accumulating studies have confirmed that autophagy is closely related to the occurrence and regulation of inflammatory diseases including IBD [12]. Autophagic genes, such as ATG16L1, ATG4, ULK1, nucleotide-binding oligomerization domain 2 (NOD2) and nuclear receptor binding factor 2 (NRBF2), have all been involved in IBD regulation; in particular, ATG16L1 and NOD2 mutations were identified to be causative of IBD in a genome-wide association study (GWAS) of IBD [13,14]. These autophagy genes were reported to regulate the function of goblet cells, increase defense ability and regulate inflammatory responses [15]. However, the principal way in which autophagy is involved in the regulation of IBD progression is still not clear.

As a canonical type of important immune cell, macrophages play an irreplaceable role in maintaining intestinal homeostasis. Polarization into the pro-inflammatory M1 subtype and the anti-inflammatory M2 subtype as the important regulatory manner of macrophage function has been intensively studied [16]. In healthy individuals, the intestinal system is protected from pathogenic microorganisms by a thick mucus layer, the tight epithelial cell barrier, and supervision of immune cells, especially macrophages, in the lamina propria [17–19]. These macrophages exhibit a highly phagocytic and anti-inflammatory phenotype [20] to clear cellular debris, dead cells, infected cells and harmless bacteria in an immune-silencing manner [21]. However, when the intestinal barrier or the pathogen-clearance pathway is destroyed, intestinal macrophages will be differentiated into an M2-like pro-inflammatory state, secrete abundant inflammatory cytokines and initiate a comprehensive immune response. Continuing exposure to microbial antigens and the uncontrolled immune response can cause further mucosal damage, altered junctional function and structure between epithelial cells, and lead to the accumulation of a large number of apoptotic cells to trigger a vicious cycle of inflammation aggravation. Therefore, enhancing the phagocytic function of macrophages to promote the clearance of pathogens and apoptotic cells is significant for inflammation alleviation and tissue repair in IBD.

The process by which phagocytic cells (mainly tissue-resident macrophages and dendritic cells) engulf dead or apoptotic cells is defined as ‘efferocytosis’. Previously, we found that NRBF2-deficient macrophages displayed impaired efferocytosis ability, which increased the susceptibility of mice to dextran sulfate sodium salt (DSS)-induced colitis [13]. In addition, many other reports have identified the critical roles of autophagic genes involved in macrophage function regulation in IBD (Table 1). These studies suggest that autophagic function in macrophages is a crucial factor in the regulation of IBD progression.

**Table 1:** Effects of autophagy-related gene mutations in macrophages on IBD

**Gene**	**Mutation site**	**Model**	**Mechanisms in macrophages**	**References**
ATG16L1	T300A	IBD patients	Influence the polarization of macrophages towards the M2 subtype; does not significantly affect NOD2 function in monocytes	[14] [39] [40]
	T300A	Atg16l1^f/f^ or colitis model mice	Enhance polyubiquitination of TRAF6 or RIPK2; induce TLR-, NLR- or IFN-β (TRIF)-mediated inflammatory signaling; impair mitophagy and MHC class II Ags processing; alter intracellular trafficking to the lysosomal compartment	[41] [42] [43]
	T316A	IBD patients	Defective clearance of the ileal pathogen *Yersinia enterocolitica* and an elevated inflammatory cytokine response	[31]
IRGM	Mutation	IBD patients	Affect autophagy resulting in inability to mediate adherent-invasive *E. coli* replication	[32,44]
NOD2	Frameshift or missense variants	IBD patients	Alter the recognition of microbial pathogens components and/or by over-activating NF-kB	[28]
		IBD patients	Affects the activation of nuclear factor NF-κB	[29]
NPC1	Mutation	IBD patients	Defective NOD2–RIPK2–XIAP pathway affects autophagosome maturation, resulting in poor elimination of intracellular bacteria	[45]
GPR65	I231L	*Citrobacter rodentium-*induced mice	Impair clearance of intracellular bacteria and accumulation of aberrant lysosomes	[36]
RNF186	rs6426833 and A64T	IBD patients	Impair E3-ubiquitin ligase activity resulting in poor clearance of intracellular bacteria	[38]
ULK-1	Mutation	IBD patients	Affect autophagy resulting in inability to mediate Adherent-Invasive *E. coli* replication	[44]
NRBF2	Deficiency	DSS-induced mice	Affect phagosome–lysosome fusion in macrophages	[13]
OPTN	rs12415716	IBD patients	Influence the release of TNF and IFN-γ	[46]
	Deficiency	Citrobacter colitis mice	Influence the release of pro-inflammatory factors	[47]
MTMR3	rs713875	IBD patients	Increase MTMR3 expression, decrease PtdIns3P and autophagy, increase caspase-1 activation	[48]
ATG5	Knocked down or knocked out	THP-1, iBMM and RAW264.7 cell lines	Cause more MIF secretion, affect the regulation of mitochondrial ROS	[49]
Atp6v0d2	Knocked out	DSS or salmonella-induced mice	Increase mitochondrial damage, enhance inflammasome activation and decrease *Salmonella typhimurium* clearance	[50]

*ATG* autophagy-related genes, *IBD* inflammatory bowel disease, *NOD2* nucleotide-binding oligomerization domain 2, *TLRs* toll-like receptors, *NLRs* NOD-like receptors, *IFN* interferon, *MHC* major histocompatibility complex, *IRGM* immunity-related GTPase family M, *NF-κB* Nuclear factor kappa-light-chain-enhancer of activated B cells, *NPC* Niemann-Pick type C, *RIPK2* receptor interacting protein kinases 2, *XIAP* X-linked inhibitor of apoptosis protein, *GPR65* G protein-coupled receptor 65, *RNF186* ring finger protein 186, *ULK-1* Unc-51 like autophagy activating kinase 1, *NRBF2* nuclear receptor binding factor 2, *OPTN* optineurin, *MTMR3* myotubulin-related protein 3, *DSS* dextran sulfate sodium salt, *MIF* macrophage migration inhibitory factor

**Figure 1:**
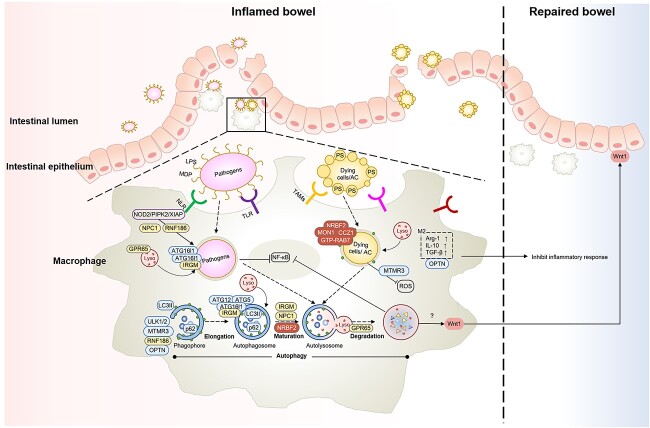
Strategies to enhance macrophage autophagy for the treatment of IBD. Macrophage autophagy at sites of inflammation in IBD can promote pathogen and apoptotic cell clearance, inflammation resolution and tissue repair. Firstly, macrophage autophagy promotes the recognition and phagocytosis of pathogens mediated by TLR, NLR and other macrophage surface receptors. Secondly, in addition to inhibiting the inflammatory response mediated by the NF-κB pathway, autophagy-related genes, such as ATG16L1, IRGM, GPR65, NPC1 and RNF186, are also involved in pathogen-containing phagosomes binding to lysosomes and degradation. Thirdly, Nrbf2 activates MON1–CCZ1–Rab7 to promote efferocytosis which induces the differentiation of macrophages into the anti-inflammatory M2 subtype. Fourthly, autophagy-activated macrophages secrete Wnt1, which promotes the repair of damaged epithelial barriers. *Lyso* lysosome, *MDP* muramyl dipeptide, *AC* apoptotic cells, *TLR* toll-like receptors, *NLR* NOD-like receptors, *ROS* reactive oxygen species, *NPC *Niemann-Pick type C

Therefore, in-depth exploration of the mechanisms of autophagy and macrophage activation in inflammation alleviation and tissue repair is beneficial to the treatment of IBD. This review discusses therapeutic strategies for promoting clearance of pathogens and apoptotic cells, tissue inflammation resolution and tissue repair by regulating autophagy in macrophages (Figure 1).

## Review

### Autophagy in IBD

There is substantial evidence linking abnormal autophagy to IBD. Autophagy-related protein Beclin-1 levels and LC3-II/I ratios in intestinal biopsy samples from IBD patients are higher than those in healthy controls and are positively correlated with disease severity [22], indicating the correlation between autophagy levels and IBD. In animals, mice with myeloid cell-specific deletion of autophagy initiation protein Atg7 are more susceptible to experimental colitis, with increased colonic cytokine expression and systemic bacterial invasion [23]. Deficiency of the autophagy gene class III PI3-kinase (*PIK3C3*) or *NRBF2* leads to IBD-like damage and pro-inflammatory responses under sterile conditions in zebrafish and mice [13,24].

To date, >150 loci have been identified to be associated with IBD based on single-nucleotide polymorphism frequency analysis in subjects with IBD vs. controls [12,25,26],including ATG16L1, immunity-related GTPase family M protein (IRGM), NOD2 and some other autophagy-related genes [27–29]. ATG16L1 is the earliest identified and most studied autophagy-related gene associated with IBD [14]. During the early stages of autophagosome maturation, ATG16L1 acts as an adaptor that stabilizes the interaction between the ubiquitin-like protein ATG12 and the E3 ubiquitin ligase-like protein ATG5, forming the ‘ATG16L complex’ with lipidated ATG8 (also known as LC3 in mammals) in an E3 ligase-like manner, which is required for autophagosome membrane elongation [30]. The T300A mutant of ATG16L1 leads to defective intestinal pathogen clearance and elevated inflammatory cytokines by reducing autophagy [31]. GWAS confirmed the association of IRGM with CD [32,33]. In humans, IRGM is a 20 kDa protein consisting of 181 amino acids expressed in the large intestine, small intestine and lymphocytes, and is involved in bacterial killing, vacuolar trafficking, lysosome acidification, phagosome maturation and virus-induced autophagy. It is also known to be involved in the control of intracellular *Mycobacterium tuberculosis* proliferation through autophagy in macrophages [34]. In addition, Saxena *et al*. [35] showed that the loss of NOD2 leads to the impairment of mitochondrial ATP synthesis and thus damages the epithelial barrier function. Lassen *et al*. [36] identified the roles of nine IBD susceptibility loci in autophagy through genomic screening, and characterized the role of GPR65 in maintaining lysosomal function and thus affecting clearance capacity and lysosome accumulation in epithelial cells and macrophages. The IBD-related gene ring finger protein 186 (RNF186) has been shown to maintain intestinal homeostasis through autophagy [37,38]. These studies demonstrate the importance of autophagy in the regulation of IBD.

At the disease level, the relationship between autophagy and IBD has been well reviewed. In short, autophagy is mainly involved in the pathogenesis and progression of IBD through enhancing xenophagy to inhibit pathogenic infection, maintaining the intestinal barrier, promoting T cell activity and proliferation, and increasing intestinal macrophage activity and inflammatory signaling pathways. Regulating the autophagy level has the potential to intervene in the IBD process from multiple aspects, such as cell function and inflammation regulation. Given the important roles of macrophages in IBD, regulating intestinal macrophage autophagy will be an important strategy for IBD treatment. Details on how macrophage autophagy affects inflammation resolution and tissue repair in IBD will be elaborated later in this review.

### Intestinal macrophages in IBD inflammation resolution and the tissue repair process

The gut is the organ in the body with direct contact with the outside. The intestinal epithelium separates the luminal contents from the body’s immune system and is an important defense barrier to protect the homeostasis of the intestinal environment and reduce inflammatory responses [51]. The intestinal immune system is mainly composed of three mucosal barrier layers, namely, the superficial mucous layer, the epithelial cell layer and the submucosa immune cell layer. The mucous layer covers the mucosal surface and insulates the epithelium from direct contact with the luminal contents by means of a gel containing various proteases. The epithelial cell layer mainly includes intestinal epithelial cells, goblet cells, Paneth cells and enteroendocrine cells that are mainly responsible for absorbing nutrients and secreting some functional proteins. The submucosa immune cell layer is composed of a variety of immune and inflammation-related cells, including macrophages, neutrophils, eosinophils and lymphocytes [52], among which macrophages are the most studied.

It is generally believed that tissue-resident macrophages in the gut originate from the monocyte system in the bone marrow [53]. Intestinal tissue has the largest population of macrophages in the body. Under physiological conditions, intestinal macrophages maintain the homeostasis of intestinal epithelial cell numbers and tissue homeostasis by phagocytosing the continuously generated naturally apoptotic epithelial cells [54,55]. Depletion of macrophages results in increased susceptibility of mice to DSS-induced colitis [56], indicating that macrophages are essential for maintaining mucosal homeostasis and protecting the body from pathogenic microorganisms *in vitro* and *in vivo* [57,58]. During inflammation, the two phenotypes of macrophages, M1 and M2, are continuously inter-switched, and an imbalance in this switch is thought to be important in the development of inflammation in IBD [59,60]. It is generally believed that at the beginning of inflammation, the number of M1 macrophages increases and that they secrete a large amount of pro-inflammatory factors such as reactive oxygen species (ROS), nitric oxide (NO), tumor necrosis factor-α (TNF-α), interleukin-6 (IL-6), IL-12 and IL-23. The secretion of IL-12 and IL-23 promotes the secretion of interferon-γ by macrophages and the recruitment of neutrophils to inflammatory sites for the purpose of inducing immunity and clearing pathogens [61]. However, sustained overexpression of M1-type responses eventually leads to destruction of the intestinal intrinsic barrier, intestinal epithelial cell apoptosis and granulation tissue hyperplasia. Furthermore, inhibition of M2 polarization leads to exacerbation of enteritis [60]. Conversely, upregulation of the expression profile of anti-inflammatory genes such as IL-4, IL-10 and TGF-β prevents excessive inflammation and promotes tissue recovery [62]. Therefore, promoting the differentiation of macrophages to the M2 anti-inflammatory phenotype is considered to be an effective approach for the treatment of IBD [63].

When suffering from invasion by endogenous or exogenous pathogens, macrophages receive ‘eat-me’ signals mainly through pattern recognition receptors (PRRs) [64], and induce adaptive immune responses by phagocytosing and presenting invading antigens to other immune cells [65]. When excessive stimuli lead to an imbalance of immune and inflammatory regulation, a large number of apoptotic cells will be observed. These apoptotic cells release ‘find-me’ and ‘eat-me’ signaling molecules, including ATP and phosphatidylserine, to recruit pro-inflammatory monocytes and macrophages to infiltrate inflamed tissues and participate in inflammation alleviation and tissue repair [66]. Insufficient clearance of apoptotic cells exacerbated the inflammatory response in a DSS-induced IBD mouse model [45], resulting in failure of tissue repair [67],whereas enhanced clearance of apoptotic cells can alleviate inflammation [68]. These results indicate that promoting the clearance of pathogens and apoptotic cells by macrophages and regulating the abnormal inflammation and immune response mediated by intestinal macrophages are important strategies for the treatment of IBD.

The uptake of apoptotic cells by macrophages (a process known as efferocytosis) is not only critical for improving the gut environment but is also an important event in phenotypic differentiation of macrophages. After recognizing and phagocytosing apoptotic cells, macrophages polarize towards the M2 phenotype, manifested by decreased expression of pro-inflammatory factors and chemokines [54,69] and increased expression of phagocytic receptors such as CD36 for further phagocytosis potential [70]. On the other hand, M2 macrophages infiltrating inflammation sites secrete signaling molecules such as soluble growth factors, polyamines and Wnt glycoproteins [71,72], which play a role in maintaining intestinal homeostasis, inhibiting inflammation and promoting tissue regeneration by acting on epithelial cells [73–76]. Ortiz-Masiá *et al*. [77] detected increased expression of Wnt1 at both the protein and mRNA levels in intestinal macrophages from IBD patients. They found that macrophages in the lamina propria express Wnt1 and the proportion of cells expressing the ligand in damaged mucosa was higher than that in normal tissue. These results indicate that polarization of macrophages to the M2 type and phagocytosis of apoptotic cells are two processes of reinforcement. During these processes, macrophages secrete a large number of cytokines, which are beneficial to inflammation regression and wound repair in IBD.

### Autophagy regulates macrophage function to promote inflammation resolution and tissue repair

#### Autophagy promotes recognition and clearance of pathogens

As mentioned earlier, macrophages need to deal with the dysfunction of inflammation resolution and fight against a large number of engulfed proteins, lipids and other degradation products under the conditions of IBD. This process has a large energy requirement. Phagocytosis and autophagy are two different modes for cells to obtain energy under nutrient-rich and nutrient-deficient conditions respectively, but they also share some pathways of material degradation to a certain extent. It is generally believed that macrophages, when subjected to pathogen invasion, enhance phagocytosis and clearance of pathogens by activating autophagy, thus freeing up more space for sustainable phagocytosis and regulating immune responses in the process.

The identification of pathogens by macrophage is mainly mediated by PRRs, such as NOD-like receptors (NLRs) in the cytoplasm and Toll-like receptors (TLRs) on the cell membrane surface, which are activated by their pathogen-associated molecular patterns [78]. NLRs and TLRs in macrophages and other innate immune cells have been shown to be closely related to autophagy, which is highly correlated with the mediation of innate immune responses in the gut [28,29]. The effect of the ATG16L1 T300A polymorphism on the TLR- or NLR-mediated signaling pathway in macrophages may be a major contributor to inflammation in CD [41]. Studies have shown that muramyl dipeptide, a component of bacterial peptidoglycan, activates NOD2 and recruits ATG16L1 to the bacterial entry sites on the plasma membrane to promote autophagy, which subsequently sequesters and kills invading *Streptococcus flexneri* and *Listeria* in autophagosomes [39,79,80]. Furthermore, macrophages with CD-associated Nod2 variants are unable to induce autophagy and had impaired ability to kill pathogenic bacteria [81]. The above results indicate that PRR achieves the regulation of autophagy in macrophages by interacting with Atg16l1. In addition, some molecules of selective autophagy were identified to be involved in selective degradation of invaders [82]. For example, the adaptor proteins p62 and NDP52 promote selective recognition and autophagy in cytoplasmic bacteria such as *Salmonella typhimurium* [83,84]. All of the above findings suggest that bacteria-induced macrophage autophagy contributes to killing invasive bacteria, and these findings are further supported by the fact that macrophage autophagy deficiency results in failure of bacterial clearance.

Autophagy is essential for macrophages to maintain their function of dealing with exogenous pathogens. Adherent-invasive *Escherichia coli* (AIEC) is a pathogenic group of bacteria isolated from the ileum of CD patients [85,86]. To date, numerous studies have revealed the active role of intestinal macrophage autophagy in maintaining recognition and responding to AIEC invasion and damage [44,87,88]. In the intestinal lamina propria, AIECs are taken up by macrophages and then survive and proliferate within macrophage vacuoles [89]. When autophagy is defective, AIECs are more likely to escape from macrophages and proliferate rapidly [44,80,88,90,91]. A study in CD patients showed that cells deficient in the autophagy-related genes IRGM and ATG16L1 increased AIEC replication [80]. Pathogen-containing vesicles were reduced in primary Atg16l1-deficient mice macrophages and ATG16L1 T300A variant human macrophages, resulting in impaired bacterial clearance and reduced major histocompatibility complex (MHC) class II antigen processing [43]. Mice deficient in Atg16l1 in bone marrow cells have increased numbers of IgA-coated gut bacteria [43]. A study by Sun *et al*. [92] identified a key role of IL-23 in promoting the clearance of bacteria by macrophages, and the performance of this function was partly related to the induction of ATG16L1 expression and autophagy.

Mechanisms of intracellular bacterial clearance barriers may be related to phagosome maturation barriers. Schwerd *et al*. [45] studied patients with NPC1-mutated Niemann-Pick type C and early-onset CD-like intestinal inflammation, showing that NOD2–RIPK2–XIAP pathway-induced antibacterial autophagy deficiency is the key cause of granulomatous intestinal inflammation, and this deficiency interferes with NOD-dependent xenophagy. Under such conditions, autophagosome maturation dysfunction, but not lysosomal dysfunction, affects autophagic elimination of intracellular bacteria, leading to dysregulated cytokine responses. Autophagy-inducing drugs can restore bacterial killing. Lipopolysaccharide (LPS), a component of the outer membrane of Gram-negative bacteria, activates TLR4 and other TLRs, thereby reducing Bcl-2 binding to Beclin 1 and increasing autophagy in macrophages [93,94]. In response to intracellular pathogens, Caspase 4 is activated, leading to inflammasome activation, which positively regulates autophagosome biogenesis and trafficking to lysosomes in macrophages, and increases efferocytosis-mediated pathogen elimination [95].

Taken together, pathogen invasion induces autophagy in macrophages. However, promoting pathogen elimination may also involve the activation of inflammatory signaling pathways. This is worth noting since it is expected to reduce pathogens and inflammation in IBD at the same time. In addition, more mechanistic studies are needed to elucidate the interplay of autophagy and inflammasome activation under different pathogen invasion conditions.

#### Autophagy promotes phagocytosis of apoptotic cells by macrophages

In recent years, efferocytosis has gradually attracted attention. When intestinal inflammation occurs, the differentiation of monocytes into mature macrophages is impaired, and the accumulation of apoptotic cells or abnormal efferocytosis causes IBD to progress to a severe degree [96,97]. Promoting efferocytosis is beneficial for the treatment of IBD. Efferocytosis directly removes apoptotic and dying cells that release inflammatory mediators, promoting the transition of macrophages to the M2 subtype [98]. Numerous studies have demonstrated the critical role of efferocytosis in atherosclerosis, cancer and various inflammatory diseases [99–101]. Mice lacking efferocytosis have accumulation of apoptotic bodies in tissues and phagocytes. A study using two different IBD mouse models confirmed that pro-catabolic factors released by efferocytic macrophages can effectively reduce intestinal inflammation and promote mucosal healing [102].

Autophagy is closely related to efferocytosis. Efferocytosis, including LC3-associated efferocytosis, shares multiple regulators with canonical autophagy, especially fusion and degradation with lysosomes [103]. Multiple studies on atherosclerosis have shown that enhancement of autophagy can induce efferocytosis of macrophages [104,105], while the absence of autophagy leads to a decrease in aortic efferocytosis [106]. Our recent study found that autophagy-related gene NRBF2-deficient mice were more susceptible to DSS-induced colitis, exhibiting more pronounced intestinal inflammation and apoptotic cell aggregation. This is due to damage of the mechanism by which NRBF2 activates the MON1–CCZ1–Rab7 module to promote the fusion of apoptotic cell-containing phagosomes and lysosomes within macrophages [13]. Until now, studies targeting the promotion of macrophage efferocytosis for the treatment of IBD have been rare. We believe that this will be a new direction for IBD treatment. However, the specific mechanism of the efferocytosis process in IBD and the exploration of efferocytosis inducers need further study.

#### Autophagy affects the anti-inflammatory activity of macrophages

Enhancing autophagy in intestinal macrophages is beneficial to the resolution of inflammation in IBD, while dysregulation of autophagy will lead to an intestinal macrophage-induced inflammatory cytokine storm and subsequent intestinal microbiota dysregulation [15]. In IBD patients, administration of the cannabinoid receptor 2 (CB2R) agonist cannabis reduces the severity of IBD by inducing autophagy and inhibiting macrophage-mediated inflammation [107]. Another study in patients showed that targeting ATG2B to inhibit autophagy enhanced the secretion of proinflammatory cytokines by macrophages and adversely affected CD [108]. In a trinitrobenzenesulfonic acid-induced IBD mouse model, the well-known anti-inflammatory cytokine IL-33 ameliorated colitis by enhancing intestinal macrophage autophagy in the inflamed gut [109]. ATG16L1 knockout mice exhibited abnormal macrophage inflammasome activity in response to TLR and TNF stimulation [42].

Several studies have shown that autophagy can achieve inflammatory suppression by promoting the polarization of macrophages towards the M2 phenotype. The activation of PI3K/Akt, an important autophagy signaling pathway, is beneficial to the differentiation of M2 macrophages and the subsequent anti-inflammatory response [110]. The use of anti-TNF antibodies [40] or induction of AMP-activated protein kinase activation [111] both achieved an increase in the proportion of M2 macrophages, resulting in an anti-inflammatory effect. In turn, loss of autophagy in macrophages promotes inflammation by increasing M1 and reducing M2 polarization [40,43]. The altered phenotype will lead to dramatic differences in the cytokines secreted by macrophages, leading to the activation of different downstream inflammation-related signaling pathways. A recent study by Gao *et al*. [41], using ATG16L1 T300A polymorphism human macrophages and Atg16L1 T300A/T300A KI mouse cells showed that autophagy impairment caused by ATG16L1 T300A polymorphism leads to increased risk of CD by inducing the canonical NFκB-mediated inflammatory pathway. Transcriptome analysis revealed that the gene encoding the selective autophagy receptor optineurin in macrophages has reduced expression in ~10% of CD patients [46]. Deletion of optineurin leads to mistransportation of cytokines such as TNF and IL6 to lysosomes, thereby reducing serum TNF levels and making mice more susceptible to *Citrobacter rodentium*-induced colitis [47]. Promotion of autophagy in macrophages inhibits CD susceptibility gene protein tyrosine phosphatase non-receptor type 2 (PTPN2)-mediated initiation and activation of the NOD-like receptor family pyrin domain containing 3 (NLRP3) inflammasome, which triggers the maturation of pro-inflammatory cytokines such as IL-1β and IL-18 thereby inhibiting colitis [112,113]. Conversely, the IBD-related risk gene myotubulin-related protein 3 increases PRR-induced IL-1β secretion and NF-κB activation by reducing autophagy in macrophages [48].

In addition to inflammatory factors, the production of ROS is also a key factor for tissue inflammation. Impaired autophagy, especially impaired mitophagy, increases ROS production, leading to tissue oxidative stress damage. Studies have shown that after LPS stimulation, human [43] or mouse [42] macrophages expressing dysfunctional ATG16L1 have impaired mitophagy, producing higher amounts of ROS and proinflammatory cytokines, impairing the processing of MHC class II antigens, altering intracellular trafficking to the lysosomal compartment and finally leading to colitis. Furthermore, macrophages depleted of LC3B, exhibited dysfunctional mitochondria associated with increased ROS production, resulting in enhanced IL1β and IL18 secretion in response to LPS [114]. Deficiency of the autophagy key gene ATG5 promotes macrophage secretion of the pro-inflammatory cytokine macrophage migration inhibitory factor, which basally responds to bacterial LPS in a mitochondrial ROS-dependent manner [49]. The macrophage-specific vacuolar-type ATPase (V-ATPase) subunit ATP6V0D2 significantly reduced the severity of DSS-induced colitis in mice by limiting inflammasome activation in macrophages and bacterial infection by pathogenic *S. typhimurium*, which was facilitated by enhanced levels of autophagosome–lysosome fusion and autophagic flux [50]. On the other hand, deficiency of the important anti-inflammatory factor IL-10, showed impaired mitophagy as well as impaired mitochondrial accumulation, increased ROS production and abnormal inflammasome activation after LPS stimulation *in vivo* and *in vitro* [115]. The IBD-related genes innate immunity activator (INAVA) [116] and autocrine/paracrine TNFSF15 [117] also affect the development of IBD by regulating ROS and autophagy pathways.

#### Autophagy is required in Wnt secretion and mucosal barrier integrity

Wnt is a secreted, lipid-modified glycoprotein. Under physiological conditions, Wnt signaling is fundamental for maintaining intestinal epithelial homeostasis and renewal. Modulation of Wnt signaling contributes to histological improvement and mucosal healing in IBD patients [118]. WNT signaling has been identified as an upstream regulator of mammalian target of rapamycin (mTOR) signaling, promoting mTORC1 activity by preventing induction of the tuberous sclerosis complex [119]. The expression of Wnt1, one of the Wnt ligands, is increased in macrophages in the damaged mucosal lamina propria, and acts on epithelial cells in a paracrine manner to activate the Wnt pathway and mediate autophagy in epithelial cells, promoting mucosal repair of damaged tissues [77].

At present, in IBD, the research about Wnt mainly focuses on fibroblasts and epithelial cells, while the relationship with macrophages is insufficiently studied. However, we can see some possible common mechanisms in other examples of inflammatory diseases. Through the study of periodontal regeneration, it has been shown that Wnt/β-catenin signaling activation can activate macrophage autophagy to promote the transformation to M2 type macrophages and achieve tissue repair [120]. Negative regulation of Wnt signaling by autophagy promotes the differentiation of Bone-marrow-derived macrophage (BMDM) into osteoclasts, thereby affecting tissue regeneration [121]. Wnt5A, a secreted Wnt glycoprotein ligand, is involved in tissue and organ morphogenesis. Wnt5A maintains cellular immune homeostasis by mediating the Rac1–NFκB pathway [122], and initiates efferocytosis to inhibit the spread of the pathogens *Pseudomonas aeruginosa* and *Streptococcus pneumoniae* [123]. IL-36γ, a member of the IL-1 superfamily, regulates autolysosome formation through non-canonical WNT signaling and the COX-2/AKT/mTOR pathway, contributing to WNT5A inhibition of intracellular *M. tuberculosis* growth [124].

In conclusion, the Wnt pathway in stimulated macrophages is related to its function in promoting tissue repair, and autophagy can regulate this activation to a certain extent. In addition, Wnt1 secreted by macrophages is beneficial for mucosal repair in epithelial cells. In-depth study of the relationship between autophagy and the Wnt pathway in macrophages will help to clarify the role of macrophages in mucosal repair.

### Clinical application of macrophage autophagy-regulating drugs in the treatment of IBD

At present, there is no approved drug to treat IBD by targeting autophagy in macrophages. However, some clinically used drugs for IBD therapy have been shown to exert mechanisms related to autophagy regulation. Anti-TNF antibodies, such as infliximab, adalimumab and golimumab, are an important therapy in IBD biological treatment [125]. Anti-TNF antibodies can induce regulatory macrophages to a phenotype similar to M2, which participate in the suppression of inflammation. The autophagy levels are closely related to phenotypic changes, where ATG16L1 and autophagy-related protein cathepsin S play a key role [40]. Prins *et al*. [126] showed that the expression level of triggering receptor expressed on myeloid cells 1 (TREM-1) in CD14+ monocytes correlates with decreased autophagy and Fc-gamma receptors (FcγR) activity, resulting in a reduced rate of differentiation into M2-type macrophages following anti-TNF monoclonal antibody (mAb) treatment, which may explain the unresponsiveness to anti-TNF therapy of IBD patients with high levels of TREM-1 expression. The combination of autophagy agonist with anti-TNF mAb may benefit the response of IBD patients to this drug.

Therapies involving modulation of autophagy and the NLRP3 inflammasome to alleviate IBD have been extensively studied. Administration of andrographolide to mice with DSS-induced colitis enhanced mitophagy in peritoneal macrophages, thereby inhibiting the NLRP3 inflammasome and subsequently ameliorating colitis [127]. Agonist-mediated activation of cannabinoid receptor 2, a G protein-coupled receptor primarily located on immune cells, that inhibits NLRP3 inflammasome activation and alleviates DSS-induced colitis in mice by increasing autophagy, has been used in clinical treatment of IBD [128].

However, the use of autophagy modulators as an IBD therapy remains challenging given the low pharmacological specificity of these drug targets, lack of specificity for cell types and autophagy-independent effects.

### Discussion

Atg16L1-, NRBF2- and ATF4-deficient or mutant mice and humans have an increased chance of developing IBD, illustrating the importance of basal autophagy in maintaining intestinal homeostasis and the intestinal defense barrier. However, hyperactivation of autophagy may aggravate IBD by inducing cell death, leading to gut barrier disruption and overproduction of proinflammatory cytokines [129–132]. Through patient samples, IBD mouse models and *in vitro* experiments, two recent studies [22,133] demonstrated that autophagy is closely related to corticotropin-releasing hormone-induced IBD, and blockade of autophagy in macrophages by chloroquine abrogates the adverse effects of corticotropin-releasing hormone. The possible reason why inhibition of autophagy is beneficial for IBD remission is that autophagy plays different roles in different disease processes. As a protective mechanism, autophagy may promote an anti-inflammatory response during the stage of inflammation, but excessive autophagy may lead to cell death under specific conditions [134]. Therefore, achieving the appropriate degree of macrophage autophagy is an important part of the treatment.

On the other hand, autophagy in different cells in the gut may have different effects on IBD development. Overall, current research suggests that autophagy by intestinal macrophages facilitates the phagocytosis of exogenous pathogenic bacteria and apoptotic cells, and promotes the repair of intestinal epithelial mucosal tissue. Increased susceptibility to IBD and loss of the mucosal barrier composed of intestinal epithelial cells have been observed in animal models with abnormal autophagy and in people with autophagy gene mutations [135]. However, autophagy in intestinal epithelial cells does not always help the body recover from IBD [136]. For example, the autophagy inhibitor chloroquine reduced the autophagic cell death of epithelial cells and attenuated the excessive inflammation it brings in a DSS-induced IBD mouse model deficient in ERBIN, which is an intestinal epithelial cell polarity-related protein [132]. Therefore, more in-depth studies are needed to distinguish the effect of cell-type-specific autophagy loss or activation on IBD outcome in different gut constituents. Drug delivery systems that precisely target and promote autophagy in intestinal macrophages may help improve the stability of drug therapy and reduce side effects.

In addition, under controlled conditions, M1 macrophages are beneficial for the destruction of exogenous pathogens, but excessive M1 activation can also produce a large number of toxic mediators to aggravate inflammation; however, M2 macrophages can balance these defects and promote tissue repair. Therefore, regulating the balance of macrophage subtypes is one of the important considerations in the treatment of IBD.

## Conclusions

It has been suggested that promoting inflammation remission and damaged tissue repair is the aim of IBD treatment and an effective way to improve the life quality of IBD patients. Growing evidence has shown a close link between macrophage autophagy and IBD in animal models and patients. In addition, recent studies have made remarkable discoveries regarding the key role of macrophage autophagy for IBD therapy: promoting pathogen and apoptotic cell clearance; activating anti-inflammatory activity; promoting tissue repair and barrier integrity. These findings open up new directions for therapeutic intervention in IBD. The next challenge in this field is how to target and controllably activate autophagy in macrophages and harness the benefits of this process for IBD therapy.

## Abbreviations

AIEC: Adherent-invasive *Escherichia coli*; Akt: Protein kinase B; ATG: Autophagy-related genes; BMDM: Bone-marrow-derived macrophage; CD: Crohn’s disease; DSS: Dextran sulfate sodium salt; FcγR: Fc-gamma receptors; GWAS: genome-wide association studies; IBD: Inflammatory bowel disease; IRGM: immunity-related GTPase family M protein; IL: Interleukin; INAVA: Innate immunity activator; LPS: Lipopolysaccharide; NF­κb: Nuclear factor kappa-light-chain-enhancer of activated B cells; NLR: NOD-like receptor; NLRP3: NOD-like receptor family pyrin domain containing 3; NOD2: Nucleotide-binding oligomerization domain 2; NPC: Niemann-Pick type C; NRBF2: Nuclear receptor binding factor 2; mAb: monoclonal antibody; mTOR: Mammalian target of rapamycin; RIPK2: Receptor interacting protein kinases 2; RNF186: Ring finger protein 186; PI3K: Phosphoinositide 3-kinases; PRR: Pattern recognition receptor; ROS: Reactive oxygen species; TLR: Toll-like receptor; TNF: Tumor necrosis factor; TREM­1: Triggering receptor expressed on myeloid cells 1; UC: Ulcerative colitis; V­ATPase: Vacuolar-type ATPase; XIAP: X-linked inhibitor of apoptosis protein.

## Funding

This study was supported by the Shenzhen Fundamental Research Program (No. SGDX20210823103804030), Science and Technology Development Fund, Macau SAR (No. 0025/2022/A1), the 2020 Guangdong Provincial Science and Technology Innovation Strategy Special Fund (Guangdong-Hong Kong-Macau Joint Lab) (No. 2020B1212030006), Guangdong Basic and Applied Basic Research Foundation (No. 2022A1515012416), National Natural Science Foundation of China (No. 31871024) and the University of Macau grants (No. MYRG2019–00129-ICMS) awarded to JHL.

## Author contribution

JHL and MYW designed the idea and framework of the review; EJW collected data and wrote the original manuscript; JHL, ZYR, YZ, RDY, CSHT and YTW participated in the review and revision of the original manuscript.

## Conflicts of interest

The authors declare no competing interests.

## Data availability

The datasets used in the present study are available from the first author and corresponding authors on reasonable request.
